# Assessment of aggressive behavior in Dravet syndrome: a critical look

**DOI:** 10.3389/fnint.2024.1403681

**Published:** 2024-04-29

**Authors:** Alejandro Torres-Fortuny, Luis Miguel Aras, Jon Andoni Duñabeitia

**Affiliations:** ^1^Asociación ApoyoDravet, Donostia-San Sebastian, Spain; ^2^Servicio Navarro de Salud-Osasunbidea, Pamplona, Spain; ^3^Centro de Investigación Nebrija en Cognición (CINC), Universidad Nebrija, Madrid, Spain

**Keywords:** Dravet syndrome, aggressiveness, antiepileptic drugs, behavior assessment, aggression

## 1 Introduction

### 1.1 Dravet syndrome

Dravet syndrome (DS) was initially described by Charlotte Dravet in 1978 as severe myoclonic epilepsy of infancy and later renamed in 1989 as Dravet syndrome (Dravet, 2011). It is a rare form of early-onset genetic epileptic syndrome characterized by refractory epilepsy and developmental delays. DS has been classified as a Developmental and Epileptic Encephalopathy (hereafter DEE), a condition associated with various comorbidities. These include intellectual disability, psychomotor delay, behavioral issues, dyssomnia, gait abnormalities, and other movement disorders such as dystonia. In DEE, cognitive development and behavior are impaired regardless of the onset of epilepsy, which is characterized by a high frequency of seizures and abundant epileptiform abnormalities (Scheffer et al., 2016, 2017; Wirrell et al., 2022).

The incidence of DS varies, with an estimated prevalence between 1 in 15,000 to 1 in 40,000 cases (Hurst, 1990; Rosander and Hallböök, 2015; Wu et al., 2015). It has been reported to affect both males and females equally (Hurst, 1990), and the first seizure usually occurs during the first year of life. Its clinical presentation also varies with age (Dravet, 2011). In addition to refractory seizures, core symptoms include developmental delays, motor dysfunction, cognitive impairment, and behavioral difficulties (Anwar et al., 2019; Jansson et al., 2020; Operto et al., 2023). These behavioral issues often encompass hyperactivity, attention deficit, mood instability, irritability, oppositional behaviors, perseverative attitudes, impulsive actions, or autism spectrum traits (Brunklaus et al., 2011; Sinoo et al., 2019; Jansson et al., 2020).

A critical aspect that represents a conundrum is that while the term “aggression” is not commonly used among the behavior difficulties most reported in the literature, recent data report the presence of aggressive behaviors in a range of 8% to 40% of patients (Wirrell et al., 2022; Postma et al., 2024). A plausible explanation for this could be that aggression-related terminology is not clearly defined and lacks universal acceptance, even among psychology or psychiatry experts. In these fields, terms such as aggression, anger, hostility, and irritability are used imprecisely and often interchangeably (Brodie et al., 2016). Additionally, when patients and caregivers report psychiatric symptoms, they sometimes do so by using informal terms that do not always align strictly with established diagnostic criteria.

The therapeutic approach in DS focuses on polytherapy, involving the use of multiple anticonvulsant medications, as well as the implementation of the ketogenic diet (Riva et al., 2022; Dini et al., 2023). Furthermore, some invasive methods, such as vagus nerve stimulation have been also proposed, although a definitive consensus on their efficacy has not been reached so far (Wirrell et al., 2022). In addition, non-pharmacological therapies have also been proposed, encompassing psychological or neuropsychological interventions, physiotherapy, sensory integration therapies such as occupational therapy, and metabolic interventions, which may include dietary adjustments and hormonal treatments (see Ballesteros-Sayas et al., 2024, for a summary). In recent years, with the introduction of new AEDs, considerable progress has been observed in seizure control in patients affected by Dravet syndrome. Undoubtedly, better control of epileptic seizures leads to a significant improvement in the quality of life for patients, families, and caregivers. However, these benefits do not always translate into an improvement in cognitive profile, and it is not strange to find cases in which effective seizure control is accompanied by increased behavioral problems, especially aggressive behaviors, which may pose a potential risk to family members and caregivers. The perplexing nature of this situation lacks a definitive explanation, and one potential contributing factor could be the adverse effects of AEDs, among other causes. The current article is set to discuss this issue in depth, paving the way for the creation of specific tools that allow for a full characterization of aggressive behavior in DS.

### 1.2 Epilepsy and aggression

The relationship between epilepsy and aggression is complex and controversial, and the literature has not yet reached definitive conclusions on this matter. As stated above, one of the plausible reasons for this is the lack of a consensual description of aggression, a term whose definition has undergone numerous iterations over time. Dollard et al. (1939, p. 11) initially defined it as an “*affectively driven attack on another with the intention of causing harm*”. Subsequently, Feshbach (1964) introduced a distinction between hostile aggression, motivated by anger, and instrumental aggression, where anger and the intention to cause harm may be absent, but harm still occurs. More recently, Bushman and Anderson (2001, p. 274) revisited the concept of the intention to cause harm, defining aggression as “*any behavior directed toward another individual that is carried out with the immediate intent of causing harm*”. Aggression is tightly linked to emotional and behavioral dysregulation. Pioneering studies reported prevalence rates of emotional and behavioral problems of up to 58.3% in children with “typical” epilepsy and some other central nervous system damage (Rutter et al., 1971). Although researchers have been investigating the relationship between epilepsy and aggressive behavior for decades, interest remains strong today due to the ongoing lack of consensus regarding the precise connections between the two (Delgado-Escueta et al., 1981; Freilinger et al., 2006; Dunn et al., 2009; Jeżowska-Jurczyk et al., 2020). Overall, a higher propensity for aggression is reported in patients with epilepsy compared to the general population (Piazzini et al., 2012). While the literature exploring aggression in Dravet syndrome is far scarcer, the aforementioned studies are also extrapolatable to DS and other DEEs, where behavioral problems, including aggressive behaviors, have also been documented (Wirrell et al., 2022; Postma et al., 2024).

Critically, the use of AEDs has been proposed as a potential trigger for the reported behavioral problems in patients with DEEs (Strzelczyk and Schubert-Bast, 2022). Unraveling the effects of AEDs on cognition, psychiatric symptoms, and behavior is a complex challenge. This is because the causes of these comorbidities are multifactorial, their manifestations are highly individual, can evolve, and may be interrelated.

Although previous studies have focused on the association between AEDs and depression or other abnormal behaviors, the specific issue of aggression in response to AEDs has long been underestimated (Brodie et al., 2016). However, it is now recognized that certain classes of medications, known for their efficacy in reducing the frequency of seizures, are associated with adverse psychiatric effects, including aggression (Dinkelacker et al., 2003; Weintraub et al., 2007; Labiner et al., 2009; Steinhoff et al., 2013; Ettinger et al., 2015; French et al., 2015). In fact, Gollan et al. (2005) have differentiated between health-related aggression and adverse effects of certain drugs, on the one hand, and deliberate or impulsive aggression, on the other, dissociating the source of the behavior depending on whether it could or not be drug-induced.

It is relevant to note that this association between AEDs and aggression has not been only described in the context of the so-considered “typical” epilepsies. As mentioned earlier, in DEE, especially in Dravet syndrome, the connection between AEDs and behavioral problems has also been reported (Strzelczyk and Schubert-Bast, 2022). DEEs are complex syndromes where the therapeutic approach often focuses on seizure control, neglecting the need to improve the identification and treatment of developmental, behavioral, and psychiatric comorbidities (including exacerbations related to treatment) (Kerr et al., 2011; Marchese et al., 2021; Samanta, 2021; Cardenal-Muñoz et al., 2022). Furthermore, a significant proportion of these patients undergo long-term polytherapy treatment with multiple AEDs instead of monotherapy, which may have a detrimental impact on higher cortical functions and exacerbate the behavioral problems.

### 1.3 Assessment of aggression and behavior

As mentioned earlier, there is a wide spectrum of definitions for the aggression construct, and consequently, dozens of scales and instruments have been developed to measure it. The selection of an instrument to assess aggression or other similar or related behavior problems should be accompanied by appropriate psychometric properties, considering its fit to the characteristics of the evaluated individual (e.g., age or cultural level), its reference group (e.g., patients with epilepsy), and the specificity of the context in which it manifests (e.g., school, work, home or social) (Carrasco and Calderon, 2006). Thus, despite the efforts put into creating specific validated tools oriented toward measuring aggressive behavior in epilepsy, these tools can hardly apply to all instances of DEE.

Specifically in the context of epilepsy, the Washington Psychosocial Seizure Inventory (WPSI) was the first questionnaire developed to evaluate psychological and social problems commonly reported in these patients (Dodrill et al., 1980). It was followed by the Walton Hospital Seizure Severity Scale (Baker et al., 1991), the Epilepsy Surgery Inventory (ESI-55) (Vickrey et al., 1992), and finally, various versions of the Quality of Life in Epilepsy (QoLIE) scale (Devinsky et al., 1995; Cramer et al., 1996, 1998) which also include specific items focusing on aggressive behavior.

Furthermore, in line with the above, the development of instruments that allow the assessment of side effects related to AEDs and patient satisfaction with the treatment has become relevant. The SEALS (Side Effect and Life Satisfaction Epilepsy Inventory) questionnaire, possibly the most widely used tool for this purpose, consists of 38 items, is easy to administer, and has been validated in various clinical trials (Gillham et al., 1996; Kane et al., 1996). However, being a self-report questionnaire, it is not useful in the context of DEEs associated with cognitive dysfunction or disabilities, and it has not been standardized for patients with intellectual deficits, as would be the case with DS. To address this gap, some instruments have been developed to assess the challenging behavior exhibited by individuals with intellectual disabilities, contributing to the development of effective intervention protocols (Reyes-Martín et al., 2022), even though these tools are not exempt from issues either.

Leaving aside the fact that some of the seemingly available instruments cannot be used freely (Einfeld and Tonge, 1995), it should be noted that most of these instruments do not consider the etiology, or degree of severity of the underlying intellectual disability (Reyes-Martín et al., 2022). This may restrict its usefulness or make its findings not applicable to other groups of patients, such as individuals with DS. In contrast, some of these instruments were developed and validated for specific syndromes or entities, such as Cornelia de Lange syndrome or individuals with autism spectrum disorders (Hyman et al., 2002; Matson et al., 2008; Frazier et al., 2023). Even some were originally designed for individuals without intellectual disabilities and later adapted to this population (Iwata et al., 1990).

Another aspect that constrains the scope of these instruments is that many of them solely assess the frequency or intensity of behaviors. However, it is also important to include variables such as duration and its impact on the environment. Addressing challenging behavior, particularly in DEE, requires an understanding of its nature and the elements that aid in comprehending and defining the behavior, including the context that interacts with it (Reyes-Martín et al., 2022).

## 2 Toward a comprehensive behavioral assessment in Dravet syndrome

Seizures constitute only one aspect of DS, which, in addition to presenting incessant epileptic activity, involves progressive brain dysfunction and behavioral problems. The combination of these elements can have a significant impact on the quality of life for caregivers (Sinoo et al., 2019; Salom et al., 2023), sometimes exceeding the impact of the seizures themselves (Kopp et al., 2008; Wheeler et al., 2008; Brunklaus et al., 2011). However, recent studies confirm the lack of adequate instruments to evaluate this impact (Sinoo et al., 2019; Gallop et al., 2021). As mentioned earlier, most studies still rely on self-report questionnaires that, although widely validated, have not been specifically developed to address this pathology (Sinoo et al., 2019; Brown et al., 2020; Jansson et al., 2020). The assessment of DS must always consider the level of intellectual disability frequently observed in these individuals. Consequently, behavioral evaluation can only be conducted through specialized questionnaires administered to family members or caregivers.

Additionally, as new AEDs emerge, the number of studies evaluating their efficacy and safety increases (Heger et al., 2020; Wirrell et al., 2022). However, there is a notable lack of attention to assessing the adverse effects on the behavior of patients with Dravet syndrome or other DEEs. Most studies on adverse events focus on more common effects on various organs or systems, overlooking psychological and behavioral effects (Nabbout et al., 2021).

Further evidence emphasizing the necessity of accurate behavioral assessment in DS is the observed underutilization of psychological services by parents, despite reported behavioral problems (Brown et al., 2020; Perinelli et al., 2023). This underutilization of available resources may result in an increased burden on patient care and a failure to apply appropriate therapies to address associated comorbidities. Early identification of psychological and behavioral disorders, coupled with the implementation of specific psychological interventions, is pivotal for delivering comprehensive care for Dravet syndrome (Brown et al., 2020). Achieving this goal necessitates the development of scales specifically designed for assessing Dravet syndrome or other DEEs.

To the best of our knowledge, the only tool specifically developed and validated for assessing DEEs is the Childhood Rare Epilepsy Social Impact Assessment (CRESIA) (Salom et al., 2022). This recently introduced tool aims to assess the social and family impact of DEEs, particularly in patients with Dravet syndrome. CRESIA encompasses assessment in six different areas, including the behavioral and emotional aspects (Salom et al., 2023). However, it is essential to note that this tool was not specifically created to dig into the potential relationship between aggressive behaviors, other behavioral issues, and factors such as the type of AEDs, polytherapy, the occurrence and severity of seizures, or the age of onset and how these aspects directly impact the quality of life of patients and caregivers.

The assessment tools of the aggression construct and other behavioral problems in DS should consider the level of intellectual disability experienced by these patients. In this context, the creation of hetero-informed scales is relevant, which, in addition to incorporating a psychometric evaluation of the patient, addresses the complexity of their situation. The importance of these aspects lies in the fact that, although the diagnosis of intellectual disability is common, the severity and course of the development of children into adulthood are unpredictable and influenced by many variables (Brunklaus et al., 2011; Lagae et al., 2018; Brown et al., 2020).

## 3 Conclusions

Addressing behavioral problems, such as aggression, in patients with DS requires a profound understanding of the complex interaction between refractory epilepsy, AEDs, developmental delay, and the individual characteristics of each patient. Advancing toward comprehensive care for patients with Dravet syndrome entails embracing multidisciplinary Approaches and developing specific tools that enable a more accurate characterization of the disease.

The different studies mentioned in this article underscore the need for developing a specific tool to assess aggressive behavior in Dravet syndrome. This is crucial for creating supportive therapies not only for patients but also for caregivers and family members. Such an approach results in a more comprehensive care strategy for individuals dealing with Dravet syndrome.

## Author contributions

AT-F: Investigation, Writing—original draft. LA: Conceptualization, Funding acquisition, Supervision, Writing—review & editing. JD: Conceptualization, Funding acquisition, Project administration, Supervision, Writing—review & editing.
